# Efficacy and Safety of Risedronate in Osteoporosis Subjects with Comorbid Diabetes, Hypertension, and/or Dyslipidemia: A Post Hoc Analysis of Phase III Trials Conducted in Japan

**DOI:** 10.1007/s00223-015-0071-9

**Published:** 2015-10-14

**Authors:** Daisuke Inoue, Ryoichi Muraoka, Ryo Okazaki, Yoshiki Nishizawa, Toshitsugu Sugimoto

**Affiliations:** Third Department of Medicine, Teikyo University Chiba Medical Center, 3426-3, Anesaki, Ichihara-shi, Chiba, 299-0111 Japan; Data Science Group, Clinical Development Department, Ajinomoto Pharmaceuticals Co., Ltd., Tokyo, Japan; Department of Metabolism, Endocrinology, and Molecular Medicine, Graduate School of Medicine, Osaka City University, Osaka, Japan; First Department of Internal Medicine, Faculty of Medicine, Shimane University, Matsue, Shimane Japan

**Keywords:** Bisphosphonate, Risedronate, Metabolic syndrome, Life style-related disease, Bone mineral density, Bone turnover markers

## Abstract

Many osteoporotics have comorbid diabetes mellitus (DM), hypertension (HT), and dyslipidemia (DL). However, whether such comorbidities alter response to anti-osteoporotic treatment is unknown. We did post hoc analyses of combined data from three risedronate Japanese phase III trials to determine whether the presence of DM, HT, or DL affects its efficacy and safety. Data from 885 subjects who received 48-week treatment with risedronate were collected and combined from the three phase III trials. They were divided into two groups by the presence or absence of comorbidities: DM (*n* = 53) versus non-DM (*n* = 832); HT (*n* = 278) versus non-HT (*n* = 607); and DL (*n* = 292) versus non-DL (*n* = 593). Bone mineral density (BMD), urinary type 1 collagen *N*-telopeptide (uNTX), and serum bone-specific alkaline phosphatase (BAP) were measured at baseline and sequentially until 48 weeks. BMD or bone markers were not different between any of the two groups. Overall, BMD was increased by 5.52 %, and uNTX and BAP were decreased by 35.4 and 33.8 %, respectively. Some bone markers were slightly lower in DM and DL subjects, but the responses to risedronate were not significantly different. Statin users had lower uNTX and BAP, but showed no difference in the treatment response. All the other medications had no apparent effect. Adverse event incidence was marginally higher in DL compared with non-DL (Relative risk 1.06; 95 % confidence interval 1.01–1.11), but was not related to increase in any specific events. Risedronate shows consistent safety and efficacy in suppressing bone turnover and increasing BMD in osteoporosis patients with comorbid DM, HT, and/or DL.

**Electronic supplementary material:**

The online version of this article (doi:10.1007/s00223-015-0071-9) contains supplementary material, which is available to authorized users.

## Introduction

Osteoporosis has become an epidemic in many aging societies including Japan. So have the components of metabolic syndrome including diabetes mellitus (DM), hypertension (HT) and dyslipidemia (DL). These metabolic diseases are not only very common, but closely related to the life style, in part mechanistically interrelated [1, 2], and may potentially affect bone metabolism. At the initiation of anti-osteoporotic treatment many patients may have such comorbidities and have been exposed to various medications for those comorbid conditions.

Epidemiological and clinical studies have established that both type 1 and type 2 DM are associated with an increased risk of fracture in part independently of bone mineral density (BMD) [3–6]. Conflicting results have been reported as to the fracture risk and BMD in subjects with HT, DL, and metabolic syndrome [7–14]. Moreover, various medications for DM, HT, or DL have been demonstrated to affect bone [15–20]. For example, it has been shown that antidiabetic thiazolidinediones increase fracture risk, whereas cholesterol-lowering statins, which inhibit 3-hydroxy-3-methylglutaryl-coenzyme A (HMG-CoA) reductase in the mevalonate pathway, and anti-hypertensive beta-blockers, which antagonize beta-adrenergic receptors, may protect bone from fractures. However, little is known whether comorbid DM, HT, DL or medications for them have any influence on the efficacy and safety of established anti-osteoporotic treatment.

In the present study, we analyzed whether comorbid DM, HT, DL, and their medications altered bone response to risedronate, a commonly prescribed oral bisphosphonate, using the data of phase III clinical trials conducted in Japan. Adverse effects were also analyzed to determine whether the comorbidities and medications affected their occurrence.

## Methods

### Data Included in the Analyses

The present analysis was conducted using the combined data from three randomized, double-blind phase III trials for risedronate [21–23], which were carried out at multiple medical institutions in Japan between March 1999 and July 2004. In the studies CCT-003 (48 weeks) and CCT-005 (96 weeks), the eligible patients were randomly assigned to receive either a daily oral dose of 2.5 mg of risedronate or an intermittent cyclical dosage regimen of etidronate consisting of cycles of 2 weeks of treatment with 200 mg/day followed by 10-week medication-free periods. In the study CCT-101 (48 weeks), the eligible patients were randomly assigned to receive either a 17.5 mg once-weekly dose or a 2.5 mg once-daily dose of risedronate. In all studies, blinding to the study drug was maintained by a double-dummy technique using active drugs and corresponding placebo tablets.

All patients were supplemented with 1.54 g calcium lactate (equivalent to 200 mg elemental calcium) throughout the study period. The daily dose of calcium was based on the result of the National Nutrition Survey conducted by the Ministry of Health, Labor and Welfare (recommended daily allowance of calcium for Japanese, 600 mg; actual intake, 585 mg on average in 1995) and the necessary amount in the elderly estimated in a calcium balance study (700–800 mg). Vitamin D was not supplemented in the three studies. Throughout the study period, concomitant use of any drugs known to affect bone metabolism was prohibited.

The study protocols were approved by the Institutional Review Board of each institution prior to initiation of the study, and all patients gave written informed consent before registration.

### Subjects

This post hoc analysis includes combined data from 885 osteoporosis patients who received treatment with risedronate for 48 weeks in the three risedronate phase III clinical trials in Japan, as described above. Ambulatory patients of either sex, aged 40–75 years in the CCT-003 trial, 50 years or older in the CCT-005 and CCT-101 trials, with involutional osteoporosis were eligible if they met the diagnostic criteria for primary osteoporosis established by the Committee of the Japanese Society for Bone and Mineral Research (JSBMR) [24, 25]. Eligible women were postmenopausal in CCT-003 and CCT-005 trials, and were at least 2 years after the last menstruation in CCT-001. Exclusion criteria included any secondary osteoporosis or other diseases known to cause reduced bone mass, any radiographic findings that might affect the vertebral integrity, recent use of drugs known to affect bone metabolism, serious renal, hepatic, or cardiac diseases, gastrointestinal diseases, drug hypersensitivity, malignant tumors under treatment with antitumor agents, history of radiotherapy to the lumbar spine or pelvis, and history of treatment with risedronate. The presence or absence of comorbid DM, HT, and DL were recorded based on attending physicians’ diagnosis at the initiation of each study. As for diabetes, subjects on insulin therapy were excluded. Thus, although precise diagnosis of type 1 or 2 DM was not made, we assume that virtually no subjects with type 1 DM should have been included.

### Endpoints

In studies CCT-003 and CCT-101, the primary efficacy endpoint was the percent change in mean L2–L4 BMD from baseline to the time of final evaluation. In CCT-005, the primary efficacy endpoint was the cumulative incidence rate of new non-traumatic vertebral fracture (including worsening of prevalent fracture) at the end of the treatment period, expressed as the percentage of patients with at least one new or worsening vertebral fracture. In the present study, the effect of risedronate on BMD, bone resorption markers [urinary *N*-terminal telopeptide of type 1 collagen (NTX) and C-terminal telopeptide of type 1 collagen (CTX)], and a bone formation marker [serum bone-specific alkaline phosphatase (BAP)] was evaluated using the combined data of the above studies.

The L2–L4 BMD was determined at baseline and after 12, 24, 36, and 48 weeks of treatment or at the time of withdrawal from the study by dual-energy X-ray absorptiometry (DXA) with the use of QDR type, XR type, or DPX type instruments in studies CCT-003 and CCT-101. Biochemical markers of bone turnover were assessed at baseline and after 4, 12, 24, 36, and 48 weeks of treatment in studies CCT-003 and CCT-101, and at baseline and 24, 48, 72, and 96 weeks in study CCT-005.

Safety was assessed by the incidence of adverse events. The objective symptoms and subjective signs related to adverse effects were monitored by noting complaints at each visit.

### Statistical Analyses

Two-sample Student’s *t* tests were conducted for comparison of age, body mass index (BMI), and baseline values and percent changes from baseline of BMD and bone markers, between groups with and without DM, HT, or DL. One-sample Student’s *t* tests were conducted for comparison of percent changes of BMD and absolute changes of NTX from baseline. One-way ANOVA was used for comparison of baseline values between groups according to the number of complications. Confidence intervals of difference between groups with and without DM, HT, or DL were determined. And confidence intervals of difference in BMD and biochemical markers of groups with one, two, and three complications were determined with the group without complications as a reference. As for longitudinal changes in BMD, interactions between time and the presence or absence of the three comorbidities were also examined by linear mixed-effect modeling. Of 885 subjects randomized and assigned to risedronate in the three studies, baseline BMD data were unavailable in CCT-005 study (*N* = 273), and CTX and BAP data were lacking in CCT-003 study (*N* = 118) and CCT-005 study (*N* = 273). Some data for BMD and biochemical markers of bone turnover at 48 weeks were missing because of early withdrawal or protocol deviations. Results are presented as means ± SD. Percent changes in BMD from the baseline were shown in boxplots expressing median and interquartile range. All the statistical analyses were done using SAS software, version 9.2 (SAS Institute Japan, Inc., Tokyo).

## Results

### Patient Characteristics at Baseline

Among the 885 subjects enrolled, those having DM, HT, and DL were 53 (6.0 %), 278 (31.4 %), and 292 (33.0 %) in number, respectively. Subjects with DM, HT, or DL were significantly older than those without (Table 1). Of the 53 patients with DM, two were taking TZD, and thirty were taking other medications for DM. The majority of patients with HT were taking calcium channel blockers (69 %), and approximately equal numbers (18 %) were taking angiotensin I converting enzyme inhibitors, angiotensin II receptor blockers, or other antihypertensives. Only 11 % were taking beta-blockers. Among 292 subjects with DL, 170 (58 %) were taking statins. There were no differences in BMD, BMI, urinary NTX or CTX, or serum BAP levels at baseline between any of the groups, with a few exceptions: Patients with DL had lower BAP, and those with DM had lower NTX and CTX, compared with those without. With respect to medications, subjects treated with statins had lower BAP (28.6 ± 9.9 vs. 31.2 ± 10.2, *p* = 0.02) and CTX (230.8 ± 104.5 vs. 265.9 ± 133.6, *p* = 0.01) compared with non-statin users. No other medications affected any bone parameters (data not shown).

**Table 1:** Baseline characteristics according to the presence or absence of comorbid diabetes, hypertension, or dyslipidemia

	Diabetes		Hypertension		Dyslipidemia	
	Yes	No	Yes	No	Yes	No
*N*	53	832	278	607	292	593
Age (years)	71.6 ± 6.6*	68.5 ± 8.1	72.1 ± 7.8*	67.1 ± 7.6	69.2 ± 8.0*	68.4 ± 8.0
Women (men)	46 (7)	807 (25)	267 (11)	586 (21)	288 (4)	565 (28)
BMI (kg/m^2^)	23.1 ± 3.2	22.1 ± 3.1	23.1 ± 3.3	21.8 ± 2.8	22.4 ± 3.0	22.1 ± 3.1
Lumbar spine BMD (g/cm^2^)	0.668 ± 0.094	0.669 ± 0.086	0.672 ± 0.090	0.668 ± 0.084	0.668 ± 0.079	0.670 ± 0.090
NTX (nmol BCE/mmol·Cr)	42.7 ± 20.3*	49.7 ± 22.8	48.9 ± 22.6	49.4 ± 22.7	48.6 ± 22.0	49.6 ± 23.0
CTX (μg/mmol·Cr)	197.1 ± 86.7*	262.5 ± 130.1	256.7 ± 115.5	259.4 ± 134.6	252.5 ± 131.2	262.2 ± 127.3
BAP (U/L)	28.2 ± 8.9	30.8 ± 10.3	31.3 ± 10.5	30.3 ± 10.0	29.5 ± 9.8*	31.4 ± 10.4

*BMI* Body mass index, *BMD* bone mineral density, *NTX* N-terminal telopeptide of type 1 collagen, *CTX* C-terminal telopeptide of type 1 collagen, *BAP* bone-specific alkaline phosphatase

* Significantly different from the group without each comorbidity (*p* < 0.05)

While almost half of the study population (46.2 %, 409/885) had no components of metabolic syndrome, 38.8 % (343/885) had one, 13.4 % (119/885) had two, and 1.6 % (14/885) had all the three (Table 2). There were no differences in BMD among groups according to the number of comorbidities. Although the number of comorbidities tended to be associated positively with age and BMI, and negatively with bone turnover markers, the trend was not statistically significant.

**Table 2:** Baseline characteristics according to the number of comorbidities

	Number of comorbidities			
	0	1	2	3
*N*	409	343	119	14
Age	67.1 ± 7.8	69.0 ± 7.9	72.7 ± 7.5	73.1 ± 7.4
Women (men)	394 (15)	331 (12)	114 (5)	14 (0)
BMI (kg/m^2^)	21.7 ± 2.8	22.3 ± 3.2	23.4 ± 3.2	22.9 ± 2.3
Lumbar spine BMD (g/cm^2^)	0.671 ± 0.089	0.663 ± 0.082	0.684 ± 0.082	0.646 ± 0.112
NTX (nmolBCE/mmol·Cr)	49.6 ± 22.5	50.0 ± 24.1	46.3 ± 17.7	45.3 ± 27.7
CTX (μg/mmol·Cr)	262.1 ± 128.6	264.0 ± 138.2	240.1 ± 98.9	193.9 ± 77.5
BAP (U/L)	31.0 ± 10.0	30.7 ± 10.7	30.1 ± 9.3	25.9 ± 8.3

*BMI* Body mass index, *BMD* bone mineral density, *NTX* N-terminal telopeptide of type 1 collagen, *CTX* C-terminal telopeptide of type 1 collagen, *BAP* bone-specific alkaline phosphatase

### Bone Mineral Density

**Fig. 1:**
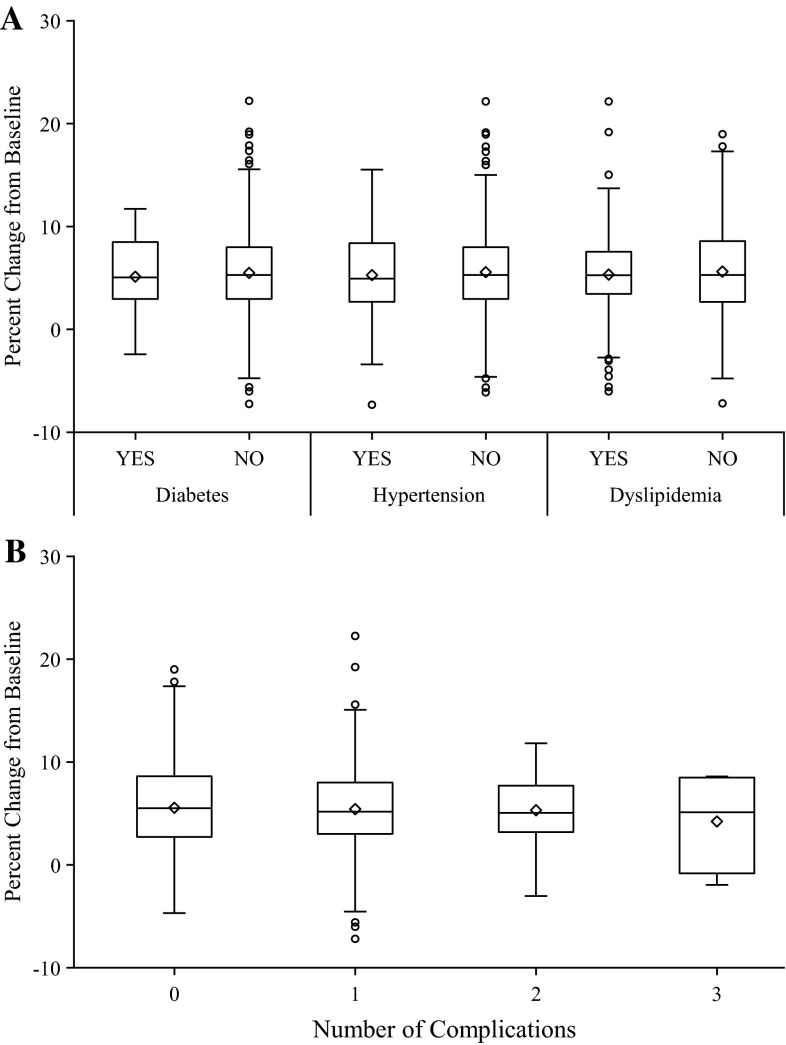
Percent changes in lumbar spine bone mineral density after 48 weeks of treatment with risedronate. The box denotes interquartile range (IQR) with the band of median inside. Outliers more than 1.5 IQR above 75th percentile or below 25th percentile are shown in *open circles*. Neither the presence or absence of each comorbid condition (**a**) nor the number of comorbidities (**b**) affected gains in bone mineral density by risedronate. *DM* diabetes, *HT* hypertension, *DL* dyslipidemia

Following 48 weeks of treatment with risedronate, a significant increase in BMD of the lumbar spine and decreases in biochemical markers of bone turnover were observed. Overall, risedronate treatment for 48 weeks resulted in 5.52 % increase in lumbar spine BMD. There was no difference in BMD gains between subjects with DM, HT, or DL and those without each comorbidity (Fig. 1a). The number of comorbidities did not influence BMD response to risedronate treatment, either (Fig. 1b). A stratified analysis by use versus non-use of treatment agents for respective metabolic disorders showed no significant difference in BMD responses to treatment with risedronate. Statin users, who had lower values of bone markers at baseline, also showed similar BMD changes as compared with statin non-users (data not shown). As for longitudinal changes in BMD at 12, 24, 36, and 48 weeks, linear mixed-effect modeling analysis also revealed no significant interaction between time and the presence or absence of each comorbidity (data not shown), indicating that none of the three comorbid conditions affected time-dependent effect of risedronate on BMD.

### Bone Turnover Markers

**Fig. 2:**
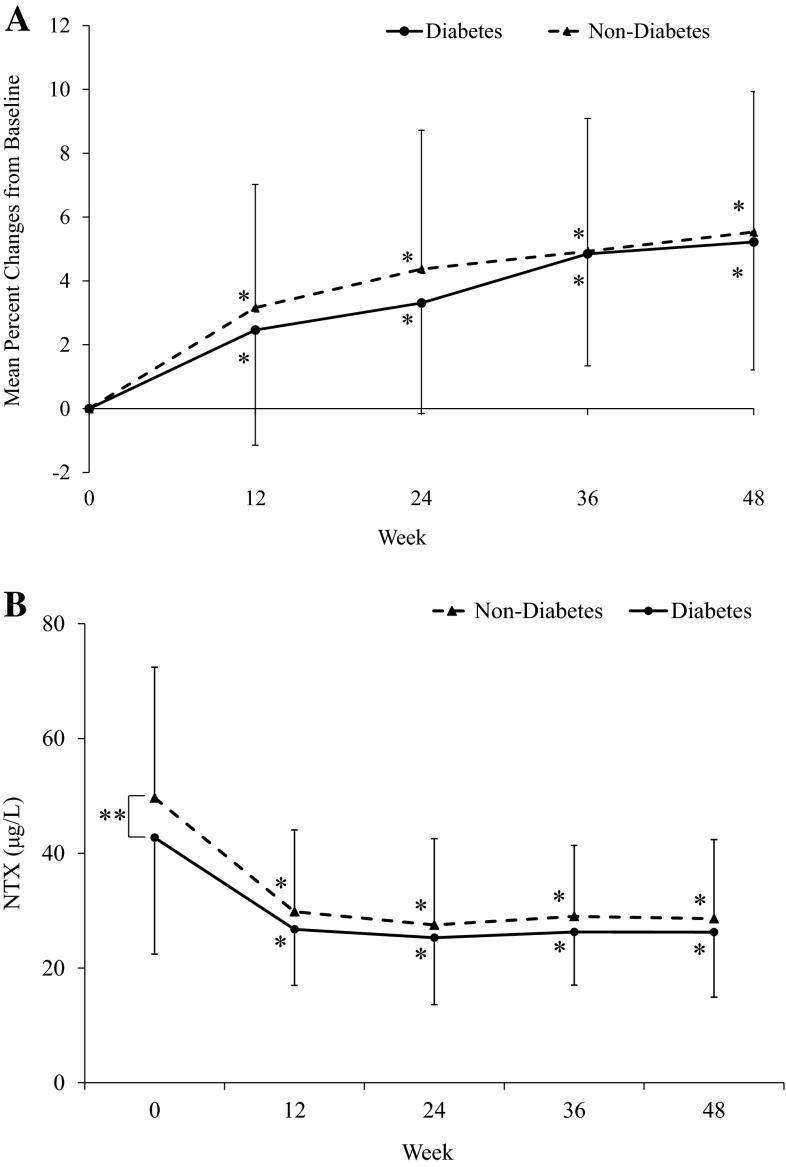
Time courses of changes in lumbar spine bone mineral density and urinary NTX excretion by risedronate treatment in subjects with or without diabetes. *Error bars* represent standard deviations. *p < 0.001, significantly different from baseline, **p < 0.05, significantly different between the two groups. **a** Bone mineral density; **b** urinary NTX

The presence or absence of each metabolic comorbidity did not affect changes in bone turnover markers in response to risedronate treatment (Table 3). The number of comorbidities also had no influence on the suppressive effect of risedronate on bone turnover markers (Table 4). In particular, although subjects with DM tended to have lower bone turnover at the baseline, treatment responses expressed as percent changes were quite similar throughout the treatment period both in terms of BMD (Fig. 2a) and bone markers (Fig. 2b). And again, no medications influenced the responses of bone turnover markers to risedronate treatment (data not shown).

**Table 3:** Percent decreases in bone turnover markers after 48 weeks of treatment with risedronate according to the presence or absence of each component of metabolic syndrome

	Diabetes		Hypertension		Dyslipidemia	
	Yes	No	Yes	No	Yes	No
NTX						
*N*	44	682	214	512	242	484
Decrease rate (%)	25.5 ± 44.0	36.0 ± 37.6	35.7 ± 44.5	35.3 ± 35.0	36.5 ± 33.7	34.8 ± 40.1
Difference	−10.5		0.4		1.7	
95 % confidence interval	−22.1–1.1		−5.6–6.5		−4.2–7.6	
CTX						
*N*	27	406	131	302	166	267
Decrease rate (%)	57.3 ± 28.9	55.1 ± 33.9	57.0 ± 25.0	54.5 ± 36.7	55.0 ± 39.5	55.4 ± 29.4
Difference	2.2		2.5		−0.4	
95 % confidence interval	−10.9–15.3		−4.4–9.4		−6.9–6.1	
BAP						
*N*	27	405	131	301	166	266
Decrease rate (%)	33.7 ± 16.7	33.8 ± 20.9	31.6 ± 20.2	34.7 ± 20.8	32.7 ± 21.5	34.4 ± 20.1
Difference	−0.0		−3.1		−1.8	
95 % confidence interval	−8.1–8.0		−7.3–1.2		−5.8–2.3	

*NTX* N-terminal telopeptide of type 1 collagen, *CTX* C-terminal telopeptide of type 1 collagen, *BAP* bone-specific alkaline phosphatase

**Table 4:** Percent decreases in bone turnover markers after 48 weeks of treatment with risedronate according to the number of comorbidities

	Number of complications			
	0	1	2	3
NTX				
*N*	341	280	95	10
Decrease rate (%)	35.8 ± 34.4	34.7 ± 43.2	36.1 ± 34.8	34.4 ± 35.9
Difference	0	−1.1	0.3	−1.4
95 % confidence interval	–	−7.3–5.0	−7.6–8.1	−23.1–20.3
CTX				
*N*	182	185	59	7
Decrease rate (%)	55.8 ± 29.4	53.4 ± 40.4	58.6 ± 21.2	64.8 ± 13.3
Difference	0	−2.4	2.8	9.0
95 % confidence interval	–	−9.7–4.9	−5.3–11.0	−13.0–31.1
BAP				
*N*	181	185	59	7
Decrease rate (%)	35.4 ± 19.5	33.0 ± 22.6	31.9 ± 17.0	29.4 ± 21.8
Difference	0	−2.4	−3.5	−6.0
95 % confidence interval	–	−6.8–2.0	−9.1–2.1	−20.8–8.9

*NTX* N-terminal telopeptide of type 1 collagen, *CTX* C-terminal telopeptide of type 1 collagen, *BAP* bone-specific alkaline phosphatase

### Adverse Event

The overall adverse event incidence was higher in the DL group compared with the non-DL group with a marginal significance (Table 5), but no specific adverse events were related to this increase in the DL group. Incidence rate of serious adverse effects was not increased in this group, either. And neither DM nor HT caused a statistically significant increase in the incidence rate of adverse events.

**Table 5:** Adverse event incidence in groups with and without diabetes, hypertension, or dyslipidemia

	Diabetes		Hypertension		Dyslipidemia	
	Yes	No	Yes	No	Yes	No
*N*	53	832	278	607	292	593
Adverse event incidence (%)	84.9	87.3	88.9	86.3	90.4	85.5
Relative risk	0.97		1.03		1.06	
95 % confidence interval	0.87–1.09		0.98–1.08		1.01–1.11	

### Vertebral Fracture

We observed a total of 33 new or worsened morphometrical vertebral fractures during 48 weeks. Of the thirty-three, fifteen were observed in patients without any comorbidities, six in patients with HT + DL, seven in patients with HT, five in patients with DL, and no patients with DM. The number of the vertebral fractures was too small to determine the influence of comorbid metabolic disorders.

## Discussion

In the present study, we demonstrated consistent efficacy of risedronate on BMD and bone turnover markers in osteoporosis patients irrespective of comorbid DM, HT, or DL. Commonly prescribed medications for those comorbidities had little effects on risedronate responsiveness. Adverse effects were also comparable among those with or without those metabolic abnormalities.

Osteoporosis, and the three diseases analyzed here, are very common among older people. Although what percentage of osteoporotics generally have DM, HT, or DL is unknown, more than half of the participants of the current study had at least one of the three comorbidities, suggesting that their coincidence is frequent. Thus, it appears clinically important to determine whether or not those comorbidities affect the effect of standard treatment modalities for osteoporosis.

### Risedronate Effect on Subjects with Metabolic Syndrome Components

The three life style-related metabolic diseases are not only prevalent all over the world, but also partly shares a common mechanism. The combination of the three is well known as metabolic syndrome, fundamentally linked to obesity and insulin resistance [1, 2, 26]. Association between metabolic syndrome and osteoporosis has been controversial [8–10]. Because insulin is considered to be bone-anabolic [27–30], it seems plausible that insulin resistance in bone [31] leads to loss of bone mass and/or qualitative integrity. However, as has been the case with type 2 DM, one reason for the difficulty in identifying potentially detrimental effects of metabolic syndrome on osteoporosis is the fact that BMD is generally positively associated with BMI: because obesity is bone-protective, it may mask the potential risk of osteoporosis associated with insulin resistance per se as well as specific pathophysiological changes caused by each component. In this sense, it seems intriguing that, in our study, subjects with metabolic syndrome components tended to have lower bone turnover, as observed with NTX in DM. Even though the clinical association of metabolic syndrome with osteoporosis and its impact on bone metabolism has not been established, our results suggest that risedronate can be safely and effectively used for the treatment of osteoporosis complicated with metabolic syndrome. As far as we know, this is the first report demonstrating that a bisphosphonate is as effective in subjects with metabolic syndrome components as in those without.

### Effect of Bisphosphonates on Diabetic Subjects

Of particular interest and importance is the effect of risedronate on subjects with DM. Clinical association between DM and fragility fracture has been established, although the mechanism is not completely understood. Subjects with type 2 DM has normal to high BMD despite increased risk of fracture, suggesting that impaired bone quality contributes to bone fragility [3–6, 29]. And some studies describe that type 2 DM is associated with decreased bone formation or low bone turnover, although conflicting results are reported [32, 33]. One may assume that these changes observed in type 2 DM could potentially blunt responses to anti-resorptive therapy, which is expected to increase BMD mainly by decreasing remodeling frequency. Contrary to such an assumption, our results clearly indicate that risedronate is equally effective in subjects with type 2 DM at least in terms of BMD gain and turnover suppression.

To date, no large-scale randomized controlled studies of any anti-osteoporotic drug efficacy on diabetic patients has been reported. The only study thus far published is the post hoc analysis of the fracture intervention trial (FIT) study, in which they examined whether the presence of diabetes affect responsiveness to alendronate, another widely used bisphosphonate [34]. This report demonstrated that, compared to the non-diabetics, diabetics lost more bone at the hip without treatment, but that the responses to alendronate in terms of BMD and bone turnover markers were similar between the two groups. These findings, as well as our current observations, strongly support an idea that the presence of diabetes has little effects on responsiveness to bisphosphonates. Collectively, these results suggest that bisphosphonates including risedronate are among the first choices for osteoporosis treatment in diabetic patients as in general population.

One important unsolved question is whether or not the same efficacy of risedronate on BMD in diabetic patients can translate into the same effect of fracture prevention. This is particularly important considering the fact that factors other than BMD, i.e., bone quality, largely contributes to the fracture risk in subjects with type 2 DM. In this sense, it is quite intriguing that fracture risk of subjects with type 2 DM has been shown to be indeed higher than that of non-diabetics at a given BMD but to be dependent on BMD: the higher BMD is, the lower the fracture risk becomes [35]. These results strongly suggest that, despite the underlying BMD-independent mechanism of osteoporosis, increasing BMD can still be an effective measure to decrease the fracture risk in subjects with type 2 DM. Definitive conclusion should await future prospective studies of fracture prevention focusing on diabetic subjects.

### Drug Interaction with Risedronate

One of the interesting findings in the present study was the fact that statin users had lower bone turnover markers at the baseline, consistent with previous reports that statins inhibit bone resorption while stimulating bone formation [36]. Although statins and bisphosphonates potentially share a common pathway in their inhibitory effect on bone resorption, our results indicate that statins do not blunt responsiveness to risedronate.

And no other drugs compromised risedronate effect in the present study. These results argue against a possibility that particular drugs affecting bone turnover may also hamper risedronate effect.

### Limitations

There are limitations in this study. First, this was a post hoc analysis of pooled data from 3 trials, which were originally neither intended to determine influence of comorbidities nor randomized for their presence. Various potential confounders may have affected the presented results. Second, the presence or absence of metabolic syndrome was not determined according to its strict criteria, as the diagnosis of metabolic syndrome components in the current study were not based on actual values of biochemical tests. Third, the number of subjects in some subgroups was small. We were therefore unable to draw definitive conclusion especially about the influence of each medication as well as the effect of risedronate in subjects with all the three comorbidities. Fourth, the current analysis did not assess risedronate effect on hip BMD. Finally, we were unable to investigate anti-fracture efficacy of risedronate due to the small sample number and the original study protocols not designed for this purpose.

## Conclusion

In summary, we demonstrated that the presence of DM, HT, or DL had virtually no effects on bone response to risedronate in subjects with osteoporosis. Commonly used medications for those comorbidities did not have apparent influence on risedronate effects, either. The profile of adverse events was also similar regardless of the presence of those metabolic diseases. We therefore conclude that physicians can prescribe risedronate to patients with those comorbidities with considerable confidence, expecting consistent efficacy and safety.

## Electronic Supplementary Material

Supplementary material 1 (docx 71 kb)

Supplementary material 2 (docx 46 kb)

## References

[1] Gallagher EJ, Leroith D, Karnieli E (2011). The metabolic syndrome: from insulin resistance to obesity and diabetes. Med Clin N Am.

[2] O’Neill S, O’Driscoll L (2015). Metabolic syndrome: a closer look at the growing epidemic and its associated pathologies. Obes Rev.

[3] Janghorbani M, Van Dam RM, Willett WC, Hu FB (2007). Systematic review of type 1 and type 2 diabetes mellitus and risk of fracture. Am J Epidemiol.

[4] Melton LJ, Leibson CL, Achenbach SJ, Therneau TM, Khosla S (2008). Fracture risk in type 2 diabetes: update of a population-based study. J Bone Miner Res.

[5] Vestergaard P (2007). Discrepancies in bone mineral density and fracture risk in patients with type 1 and type 2 diabetes: a meta-analysis. Osteoporos Int.

[6] Yamamoto M, Yamaguchi T, Yamauchi M, Kaji H, Sugimoto T (2007). Bone mineral density is not sensitive enough to assess the risk of vertebral fractures in type 2 diabetic women. Calcif Tissue Int.

[7] Cui LH, Shin MH, Chung EK, Lee YH, Kweon SS, Park KS, Choi JS (2005). Association between bone mineral densities and serum lipid profiles of pre- and post-menopausal rural women in South Korea. Osteoporos Int.

[8] Ahmed LA, Schirmer H, Berntsen GK, Fonnebo V, Joakimsen RM (2006). Features of the metabolic syndrome and the risk of non-vertebral fractures: the Tromso study. Osteoporos Int.

[9] Dennison EM, Syddall HE, Aihie Sayer A, Martin HJ, Cooper C (2007). Lipid profile, obesity and bone mineral density: the hertfordshire cohort study. QJM.

[10] von Muhlen D, Safii S, Jassal SK, Svartberg J, Barrett-Connor E (2007). Associations between the metabolic syndrome and bone health in older men and women: the Rancho Bernardo study. Osteoporos Int.

[11] Vestergaard P, Rejnmark L, Mosekilde L (2009). Hypertension is a risk factor for fractures. Calcif Tissue Int.

[12] Yamaguchi T, Kanazawa I, Yamamoto M, Kurioka S, Yamauchi M, Yano S, Sugimoto T (2009). Associations between components of the metabolic syndrome versus bone mineral density and vertebral fractures in patients with type 2 diabetes. Bone.

[13] Ilic K, Obradovic N, Vujasinovic-Stupar N (2013). The relationship among hypertension, antihypertensive medications, and osteoporosis: a narrative review. Calcif Tissue Int.

[14] Lee SH, Baek S, Ahn SH, Kim SH, Jo MW, Bae SJ, Kim HK, Choe J, Park GM, Kim YH, Koh JM, Kim BJ, Kim GS (2014). Association between metabolic syndrome and incident fractures in Korean men: a 3-year follow-up observational study using national health insurance claims data. J Clin Endocrinol Metab.

[15] Bauer DC, Mundy GR, Jamal SA, Black DM, Cauley JA, Ensrud KE, van der Klift M, Pols HA (2004). Use of statins and fracture: results of 4 prospective studies and cumulative meta-analysis of observational studies and controlled trials. Arch Intern Med.

[16] Reid IR, Gamble GD, Grey AB, Black DM, Ensrud KE, Browner WS, Bauer DC (2005). Beta-blocker use, BMD, and fractures in the study of osteoporotic fractures. J Bone Miner Res.

[17] Nguyen ND, Wang CY, Eisman JA, Nguyen TV (2007). On the association between statin and fracture: a Bayesian consideration. Bone.

[18] Monami M, Cresci B, Colombini A, Pala L, Balzi D, Gori F, Chiasserini V, Marchionni N, Rotella CM, Mannucci E (2008). Bone fractures and hypoglycemic treatment in type 2 diabetic patients: a case-control study. Diabetes Care.

[19] Loke YK, Singh S, Furberg CD (2009). Long-term use of thiazolidinediones and fractures in type 2 diabetes: a meta-analysis. CMAJ.

[20] Kanazawa I, Yamaguchi T, Yamamoto M, Sugimoto T (2010). Relationship between treatments with insulin and oral hypoglycemic agents versus the presence of vertebral fractures in type 2 diabetes mellitus. J Bone Miner Metab.

[21] Fukunaga M, Kushida K, Kishimoto H, Shiraki M, Taketani Y, Minaguchi H, Inoue T, Morita R, Morii H, Yamamoto K, Ohashi Y, Orimo H (2002). A comparison of the effect of risedronate and etidronate on lumbar bone mineral density in Japanese patients with osteoporosis: a randomized controlled trial. Osteoporos Int.

[22] Kishimoto H, Fukunaga M, Kushida K, Shiraki M, Itabashi A, Nawata H, Nakamura T, Ohta H, Takaoka K, Ohashi Y (2006). Efficacy and tolerability of once-weekly administration of 17.5 mg risedronate in Japanese patients with involutional osteoporosis: a comparison with 2.5-mg once-daily dosage regimen. J Bone Miner Metab.

[23] Kushida K, Fukunaga M, Kishimoto H, Shiraki M, Itabashi A, Inoue T, Kaneda K, Morii H, Nawata H, Yamamoto K, Ohashi Y, Orimo H (2004). A comparison of incidences of vertebral fracture in Japanese patients with involutional osteoporosis treated with risedronate and etidronate: a randomized, double-masked trial. J Bone Miner Metab.

[24] Orimo H, Hayashi Y, Fukunaga M, Sone T, Fujiwara S, Shiraki M, Kushida K, Miyamoto S, Soen S, Nishimura J, Oh-Hashi Y, Hosoi T, Gorai I, Tanaka H, Igai T, Kishimoto H (2001). Diagnostic criteria for primary osteoporosis: year 2000 revision. J Bone Miner Metab.

[25] Orimo H, Sugioka Y, Fukunaga M, Muto Y, Hotokcbuchi T, Gorai L, Nakamura T, Kushida K, Tanaka H, Ikai T, Ohhashi Y (1998). Diagnostic criteria of primary osteoporosis. J Bone Miner Metab.

[26] Bassi N, Karagodin I, Wang S, Vassallo P, Priyanath A, Massaro E, Stone NJ (2014). Lifestyle modification for metabolic syndrome: a systematic review. Am J Med.

[27] Ferron M, Wei J, Yoshizawa T, Del Fattore A, DePinho RA, Teti A, Ducy P, Karsenty G (2010). Insulin signaling in osteoblasts integrates bone remodeling and energy metabolism. Cell.

[28] Fulzele K, Riddle RC, DiGirolamo DJ, Cao X, Wan C, Chen D, Faugere MC, Aja S, Hussain MA, Bruning JC, Clemens TL (2010). Insulin receptor signaling in osteoblasts regulates postnatal bone acquisition and body composition. Cell.

[29] Hamann C, Kirschner S, Gunther KP, Hofbauer LC (2012). Bone, sweet bone—osteoporotic fractures in diabetes mellitus. Nat Rev Endocrinol.

[30] Klein GL (2014). Insulin and bone: recent developments. World J Diabetes.

[31] Wei J, Ferron M, Clarke CJ, Hannun YA, Jiang H, Blaner WS, Karsenty G (2014). Bone-specific insulin resistance disrupts whole-body glucose homeostasis via decreased osteocalcin activation. J Clin Investig.

[32] Shu A, Yin MT, Stein E, Cremers S, Dworakowski E, Ives R, Rubin MR (2012). Bone structure and turnover in type 2 diabetes mellitus. Osteoporos Int.

[33] Starup-Linde J, Eriksen SA, Lykkeboe S, Handberg A, Vestergaard P (2014). Biochemical markers of bone turnover in diabetes patients: a meta-analysis, and a methodological study on the effects of glucose on bone markers. Osteoporos Int.

[34] Keegan TH, Schwartz AV, Bauer DC, Sellmeyer DE, Kelsey JL (2004). Effect of alendronate on bone mineral density and biochemical markers of bone turnover in type 2 diabetic women: the fracture intervention trial. Diabetes Care.

[35] Schwartz AV, Vittinghoff E, Bauer DC, Hillier TA, Strotmeyer ES, Ensrud KE, Donaldson MG, Cauley JA, Harris TB, Koster A, Womack CR, Palermo L, Black DM, Study of Osteoporotic Fractures Research G, Osteoporotic Fractures in Men Research G, Health A, Body Composition Research G (2011). Association of BMD and FRAX score with risk of fracture in older adults with type 2 diabetes. JAMA.

[36] Chuengsamarn S, Rattanamongkoulgul S, Suwanwalaikorn S, Wattanasirichaigoon S, Kaufman L (2010) Effects of statins vs. non-statin lipid-lowering therapy on bone formation and bone mineral density biomarkers in patients with hyperlipidemia. Bone 46(4):1011–1015. doi:10.1016/j.bone.2009.12.023 10.1016/j.bone.2009.12.023 20045497

