# Supplementary material for: Efficacy and Safety of Risedronate in Osteoporosis Subjects with Comorbid Diabetes, Hypertension, and/or Dyslipidemia: A Post Hoc Analysis of Phase III Trials Conducted in Japan
Source: Calcif Tissue Int. 2015 Oct 14;98:114–22. doi: 10.1007/s00223-015-0071-9 (PMC4723633; doi:10.1007/s00223-015-0071-9)
Supplement: Supplementary file 1 — Supplementary material 1 (docx 71 kb) [file 223_2015_71_MOESM1_ESM.docx]

Supplement Table 2. Linear mixed-effect modeling analysis.

Type III SS test of fixed effect (Diabetes)

| effect | df of numerator | df of denominator | F value | Pr > F |
| --- | --- | --- | --- | --- |
| DM yes/no | 1 | 2152 | 0.06 | 0.8029 |
| Time | 4 | 2152 | 74.22 | <.0001 |
| DM yes/no * Time | 4 | 2152 | 0.71 | 0.5871 |

Type III SS test of fixed effect (Hypertension)

| effect | df of numerator | df of denominator | F value | Pr > F |
| --- | --- | --- | --- | --- |
| HT yes/no | 1 | 2152 | 0.97 | 0.3258 |
| Time | 4 | 2152 | 307.42 | <.0001 |
| HT yes/no * Time | 4 | 2152 | 0.45 | 0.7729 |

Type III SS test of fixed effect (Dyslipidemia)

| effect | df of numerator | df of denominator | F value | Pr > F |
| --- | --- | --- | --- | --- |
| DL yes/no | 1 | 2152 | 0.61 | 0.4360 |
| Time | 4 | 2152 | 333.52 | <.0001 |
| DL yes/no * Time | 4 | 2152 | 2.19 | 0.0676 |

As shown above, each comorbid condition did not show significant interaction with time (12, 24, 36, 48 wk) in terms of risedronate effect on BMD.
