# Supplementary material for: Efficacy and Safety of Risedronate in Osteoporosis Subjects with Comorbid Diabetes, Hypertension, and/or Dyslipidemia: A Post Hoc Analysis of Phase III Trials Conducted in Japan
Source: Calcif Tissue Int. 2015 Oct 14;98:114–22. doi: 10.1007/s00223-015-0071-9 (PMC4723633; doi:10.1007/s00223-015-0071-9)
Supplement: Supplementary file 2 — Supplementary material 2 (docx 46 kb) [file 223_2015_71_MOESM2_ESM.docx]

Supplement Table 1. Estimated detectable percent differences between those with and without each comorbidity.

BMD（Fig 1A）

|  | Diabetes | Hypertension | Dyslipidemia |
| --- | --- | --- | --- |
| 48 wk | 2.3 | 1.2 | 1.1 |

BMD（Fig 1B）

|  | 1 | 2 | 3 |
| --- | --- | --- | --- |
| 48 wk | 1.2 | 1.5 | 5.4 |

BMD（Fig. 2A）

|  | Diabetes |
| --- | --- |
| 12 wk | 1.9 |
| 24 wk | 1.9 |
| 36 wk | 2.0 |
| 48 wk | 2.3 |

NTX（Fig. 2B）

|  | Diabetes |
| --- | --- |
| 0 wk | 8.3 |
| 12 wk | 5.2 |
| 24 wk | 5.2 |
| 36 wk | 5.0 |
| 48 wk | 5.1 |
